# Multilocus sequence typing (MLST) of clinical and environmental isolates of *Cryptococcus neoformans* and *Cryptococcus gattii* in six departments of Colombia reveals high genetic diversity

**DOI:** 10.1590/0037-8682-0422-2019

**Published:** 2020-09-11

**Authors:** Norida Vélez, Patricia Escandón

**Affiliations:** 1 Grupo de Microbiología, Instituto Nacional de Salud, Bogotá, Colombia.

**Keywords:** Cryptococcus neoformans, Cryptococcus gattii, Multilocus Sequence Typing, Incidence, Colombia

## Abstract

**INTRODUCTION:**

The average annual incidence of cryptococcosis in Colombia is 0.23 cases per 100,000 inhabitants in the general population, and 1.1 cases per 1000 in inhabitants with Acquired Immune Deficiency Syndrome (AIDS). In addition, the causal fungus has been isolated from the environment, with serotypes A-B and C in different regions. This study aims to determine the genetic association between clinical and environmental isolates of *C. neoformans/C. gattii* in Colombia.

**METHODS:**

Multilocus sequence typing (MLST) was used to identify possible clones, providing information about the epidemiology, ecology, and etiology of this pathogen in Colombia.

**RESULTS:**

A total of 110 strains, both clinical (n=61) and environmental (n=49), with 21 MLST sequence types (ST) of *C. neoformans* (n=14STs) and *C. gattii* (n=7STs) were identified. The STs which shared clinical and environmental isolate sources were grouped in different geographical categories; for *C. neoformans*, ST93 was identified in six departments, ST77 in five departments; and for *C. gattii*, ST25 was identified in three departments and ST79 in two.

**CONCLUSIONS:**

High genetic diversity was found in isolates of *C. neoformans/gattii* by MLST, suggesting the presence of environmental sources harboring strains which may be sources of infection for humans, especially in immunocompromised patients; these data contribute to the information available in the country on the distribution and molecular variability of *C. neoformans* and *C. gattii* isolates recovered in Colombia.

## INTRODUCTION

Cryptococcosis is a fungal disease of worldwide distribution. Patients acquire the infection by exposure and inhalation of fungal propagules present in environmental sources. This infection is considered potentially fatal, and affects the lungs and the central nervous system^1^ ^-^ ^3^in both immunosuppressed individuals and in those with an apparently intact immune system^4^ ^,^ ^5^. In Colombia, the annual incidence for this infection is 0.23 cases per 100.000 inhabitants in the general population and 1.1 cases per 1000 inhabitants in Acquired Immune Deficiency Syndrome (AIDS) patients (period 1997-2013)^3^. 

Although different taxonomic classifications have been proposed to categorize the etiological agent of the disease, Hagen et al. have suggested that different molecular types should be considered as independent species^6^. This suggestion has not been fully accepted by the scientific community^7^. In the present investigation, we refer to isolates as the *C. neoformans* species complex and the *C. gattii* species complex^7^. Cryptococcosis is caused by the *C. neoformans* species complex and the *C. gattii* species complex^7^. The first species consists of two varieties, *C. neoformans* var. *grubii* (serotype A) and *C. neoformans* var. *neoformans* (serotype D). In addition to the hybrid AD serotype, the species has a worldwide distribution and preferentially affects immunocompromised individuals, mainly those infected with the human immunodeficiency virus (HIV)^2^ ^-^ ^4^. In the environment, it has been associated with bird excreta, especially from soils contaminated with pigeon (*Columba livia*) droppings^8^. The second causative agent of cryptococcosis, the *C. gattii* species complex, comprises serotypes B and C, which can be found in the environment in decaying plant material (hollows, leaf, bark, flowers, soil, fruit) from different trees (*Eucalyptus* spp., acacias, *Ficus* spp., and *Terminalia catappa*) in various regions of the world^9^ ^,^ ^10^. It is found mainly in tropical, subtropical, and temperate regions^11^ ^-^ ^13^. Currently, several interspecies hybrids have been described between serotypes BD and AB^2^ ^,^ ^14^.

Many molecular techniques have been applied in the epidemiological study of *C. neoformans* and *C. gattii* isolates. The most common techniques are PCR fingerprinting^15^, restriction fragment length polymorphism (RFLP) of the *PLB1* and *URA5* genes^15^, amplified fragment length polymorphism (AFLP)^16^, and the most recently developed technique of multilocus sequence typing (MLST)^17^ ^,^ ^18^. For genotyping *Cryptococcus* species using MLST, six conserved genes (*CAP59*, *GPD1*, *LAC1*, *PLB1*, *SOD1* and *URA5*) and the intergenic region IGS1 are used. MLST has a high discriminatory power for the genotyping of isolates to determine clonality. It is also highly discriminatory for a large number of pathogens. MLST directly measures changes in the sequence of a series of conserved genes, characterizes isolates by allelic profiles, and is an excellent tool for taxonomic characterization at the molecular level^17^. However, more robust techniques such as whole genome sequencing (WGS) allow for the detection of differences between the molecular types at the genomic level^19^. In Colombia, studies have been carried out describing the importance and potential relationship between clinical and environmental isolates using molecular typing techniques such as PCR fingerprinting and *URA5*-RFLP^10^ ^,^ ^15^ ^,^ ^20^, and have contributed to the knowledge about the epidemiology of these pathogens. 

Although the results from previous studies are very important and have contributed to the knowledge about the epidemiology of these pathogens, the genetic diversity of the Colombian isolates is suspected to be more diverse. Therefore, this study aims to determine the genetic relationship between clinical and environmental isolates of the *C. neoformans* species complex and the *C. gattii* species complex in Colombia using MLST. In addition, identifying possible clones and a more precise association between clinical and environmental strains will provide important information about the epidemiology, ecology, and etiology of this pathogen in Colombia.

## METHODS

### Study areas and biological material

A set of 88 *C. neoformans* species complex isolates (clinical: n=47; environmental: n=41) and 22 *C. gattii* species complex isolates (clinical: n=14; environmental: n=8) were included. These isolates which were recovered between 2005-2014 were available in the strain bank of the Microbiology Group at the Instituto Nacional de Salud, recovered in the departments of Antioquia (18.3%), Atlántico (14.6%), Bogotá DC (15.6%), Cauca (13.7%), Norte de Santander (20.8%), and Valle (17.4%), and were previously typed by PCR fingerprinting or RFLP of the *URA5* gene. Isolates were preserved in 10% glycerol at -70 °C. Of these isolates, 86 (78%) belonged to *C. neoformans* molecular type VNI, two (1.8%) belonged to *C. neoformans* molecular type VNII; four (3.6 %) isolates belonged to *C. gattii* molecular type VGI, 10 (9.2%) belonged to VGII, and 8 (7.3%) belonged to the VGIII molecular type. From these, 12 *C. gattii* isolates of clinical origin were previously typed by Lizarazo J, et al. in 2014^21^. Thus, a total of 110 isolates were studied ( Supplementary material 1).

### Patient data

Of a total of 61 clinical isolates (*C. neoformans*: n=47; *C. gattii*: n=14), 78.3% were isolated from men. The mean age was 42 years with a minimum of 18 years and a maximum of 82 years. The most common symptom was headache in 69.6% of the cases, followed by fever and nausea in 56.5%, while confusion, visual alterations, cough, and weight loss were also observed in a small percentage of patients; 70.4% of the patients presented with at least one risk factor, with HIV/AIDS being the most common. Of these cases, 16.6% were diagnosed concurrently with AIDS and cryptococcosis. A total of 88.3% were new cases, and 11.2% were relapses; 23.0% of the patients died ( Supplementary material 2a). 

### Environmental data

Forty-nine environmental isolates were selected (*C. neoformans:* n=41; *C. gattii*: n=8); 73.5% (n=36) were recovered from 10 different types of trees, and 26.5% (n=13) were recovered from *Columba livia* droppings ( Supplementary material 2b).

### Molecular Analysis

a) Genomic DNA extraction was performed as previously described by Casali A, et al. (2003)^22^. Briefly, *C. neoformans* and *C. gattii* were plated on yeast extract-peptone-dextrose (YEPD) agar for 48 hours at 27 °C; 10 μl of yeast cells was placed in an Eppendorf tube using an inoculation loop, and incubated at -20 °C for one hour. The cells were then suspended in 500 μl of lysis buffer (10 mM Tris, pH 7.5, 1 mM EDTA, pH 8.0, and 1% SDS) and incubated at 65 °C for one hour; 500 μl of phenol:chloroform:isoamyl alcohol (25:24:1) was added, and the sample was centrifuged for 15 minutes at 13,000 rpm. The supernatant was transferred to a new tube, and an equal volume of isopropanol was added. The DNA was precipitated at -20 °C for one hour, and centrifuged for 15 minutes at 4 °C at 13,000 rpm. The DNA was then precipitated with 70% ethanol and centrifuged again for 15 minutes at 4 °C at 13,000 rpm, and subsequently dried at room temperature. The samples were resuspended in 5 µl Tris-EDTA (TE) buffer and stored at 4 °C.

b) MLST typing: Typing of the isolates was performed using the International Society for Human and Animal Mycology (ISHAM) consensus MLST scheme of seven genetic loci: *CAP59, GPD1,* IGS1*, LAC1, PBL1, SOD1,* and *URA5* ^17^, with minor modifications. Individual PCRs were performed in a final volume of 20 μl and a volume of 2 μl of DNA was added at a concentration of 1 ng/µl for the amplification of *CAP59*, *SOD1*, IGS1, and *GPD1,* and a volume of 5 µl of DNA at a concentration of 5 ng/µl for amplifying the *LAC1*, *PLB1,* and *URA5* genes. 

PCR products were purified and sequenced commercially by the sequencing service provider Macrogen, Inc. Sequences were analyzed using the Sequencher Software 5.2 (Gene Codes Corporation, MI, USA). Six reference strains were used: WM148 (VNI-CBS10085), WM626 (VNII-CBS10086), WM179 (VGI-CBS10078), WM178 (VGII-CBS10082), WM175 (VGIII-CBS10081), and WM779 (VGIV-CBS10101)^15^. Dendrograms were created with the Mega 5.0 software, using the individual locus sequences and the concatenated sequences^23^. The evolutionary history was derived using the maximum likelihood method based on the Jukes-Cantor model, and bootstrap values were displayed for each branch (1000 repetitions). Allele types and combined sequence types were assigned using the ISHAM consensus database^24^. The data were tabulated using Microsoft Excel®. Additionally, *C. gattii* sequences reported previously by Lizarazo J, et al. in 2014^21^ were included to increase the robustness of the analysis. 

Genetic diversity of isolates was determined by using the DnaSP v5 software; this variability was extracted from concatenated sequences associated with genes, department, molecular type, and origin (clinical or environmental)^25^, to detect genetic polymorphism levels. The distribution was determined by calculating the haplotype (gene) diversity, nucleotide diversity (π) (the average number of nucleotide differences per site between two sequences), and θ indexes (per site, as an indicator of mutation rate per nucleotide site per generation), calculated from Eta (h) (the total number of mutations and “S”, the number of segregating/polymorphic sites). Each index was reported with the corresponding standard deviation. The π indexes for each set of data were compared to identify the category with the greatest diversity.

## RESULTS

**FIGURE 1:**
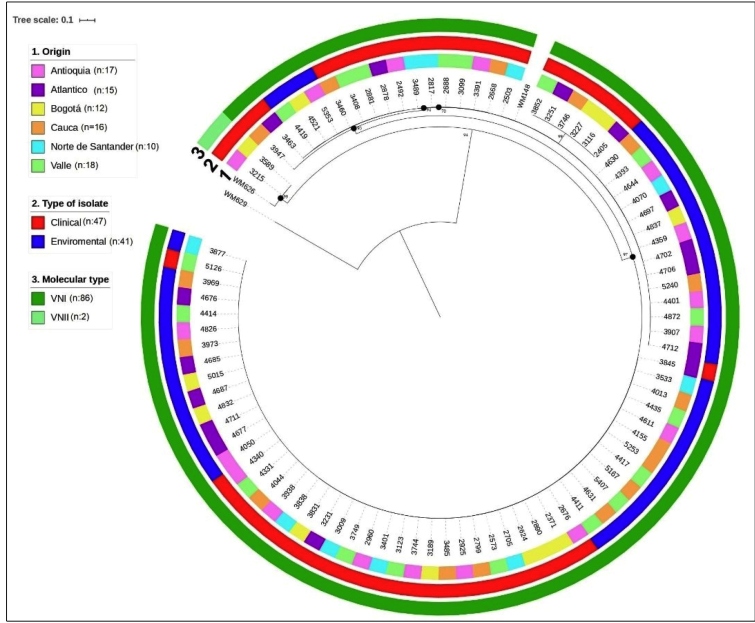
Phylogenetic analysis of 88 clinical and environmental isolates of *C. neoformans*. The evolutionary history was derived using the maximum likelihood method based on the Jukes-Cantor model using concatenated nucleotide sequences of 7 loci and a representative for each multilocus sequence typing (MLST) sequence type. Bootstrap values are shown for each branch (1000 repetitions).

**FIGURE 2:**
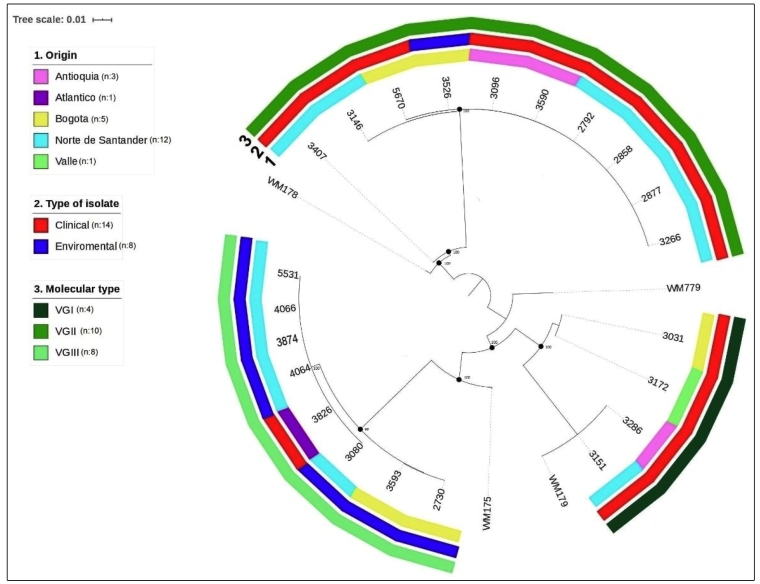
Phylogenetic analysis of 21 clinical and environmental isolates of *C. gattii*. The evolutionary history was derived using the maximum likelihood method based on the Jukes-Cantor model using concatenated nucleotide sequences of 7 loci, and a representative for each multilocus sequence typing (MLST) sequence type. Bootstrap values are shown for each branch (1000 repetitions).

A total of 98 isolates were typed by MLST (*C. neoformans*: n=88; *C. gattii*: n=10); additionally, 12 clinical isolates of *C. gattii* sequences reported previously by Lizarazo J, et al. in 2014 were included^21^. Twenty-one STs were identified, and 13 STs were assigned to the molecular type VNI and one ST to the *C. neoformans* molecular type VNII; three STs were assigned to molecular type VGII, and two STs each were assigned to the VGI and VGIII molecular types (Table 1). The genetic associations among the isolates for *C. neoformans* and *C. gattii* are shown in Figures 1 and 2, respectively. The MLST data of sequences of identified alleles were deposited in the GenBank database ( Supplementary material 3).

**TABLE 1:** Sequence types of *Cryptococcus neoformans* and *Cryptococcus. gattii* in clinical and environmental isolates from Colombia.

		Departments							
Molecular type	ST	Antioquia	Atlántico	Bogotá	Cauca	Nte. Santander	Valle	Total
**Environmental**								
VNI	15	-	-	1	-	-	1	2
	23	4	4	1	1	1	2	13
	56	-	-	-	1	-	-	1
	77	1	-	-	5	1	4	11
	93	2	5	2	2	1	1	13
	226	1	-	-	-	-	-	1
VGII	25	-	-	1	-	-	-	1
VGIII	75	-	-	2	-	-	-	2
	79	-	-	-	-	5	-	5
								
**Clinical**								
VNI	2	-	2	2	1	-	1	6
	5	1	1	-	1	-	2	5
	6	-	1	-	-	-	-	1
	63	-	-	-	-	1	-	1
	69	1	-	-	1	1	2	5
	71	-	-	-	-	1	-	1
	77	-	1	-	-	-	-	1
	93	6	1	5	3	4	5	24
	532	-	-	-	1	-	-	1
VNII	40	1	-	1	-	-	-	2
VGI	51	1	-	-	-	1	-	2
	58		-	1	-	-	1	2
VGII	25	2	-	1	-	4	-	7
	323	-	-	-	-	1	-	1
	324	-	-	-	-	1	-	1
VGIII	79	-	1	-	-	-	-	1
	**Total**	20	16	17	16	22	19	110

**ST:** sequence type.

In 88 clinical and environmental isolates of *C. neoformans,* 14 different STs were identified, the most frequent of which was ST93 (42%), followed by ST23 (14.7%), ST77 (13.6%), ST2 (6.8%), ST5, ST6, ST15, ST40, ST56, ST63, ST69, ST71, and ST226. ST532, a novel *C. neoformans* ST was identified, and this is the first report of this ST worldwide. Of the 22 isolates of *C. gattii,* seven different STs were identified; the most frequent ST was ST25 (36.3%), followed by ST79 (27.2%) and, in lesser proportions, ST51, ST58, ST75, ST323, and ST324. Table 1 shows the STs found in both the clinical and environmental isolates.

Diversity indexes were also calculated per species, department, origin, and molecular type. It was found that the haplotype diversity in *C. neoformans* was 14 and in *C. gattii* it was 11; the nucleotide diversity index was higher in *C. gattii* (Pi= 0.868) than in *C. neoformans* (Pi= 0.779) ( Supplementary material 3). The diversity index calculated by departments showed that Cauca and Valle presented with greater diversity of haplotypes for *C. neoformans* (Hd=0.833 and 0.8443, respectively), and Bogotá and Norte de Santander for *C. gattii* (Hd=0.8 and 0.818 respectively). The diversity by type of origin (clinical or environmental) did not vary between the two species. The haplotypic diversity for *C. gattii* was higher in VGI and VGIII (Hd= 0.667 and 0.607 respectively), when compared to VGII (Hd= 0.378) ( Supplementary material 4).

**FIGURE 3:**
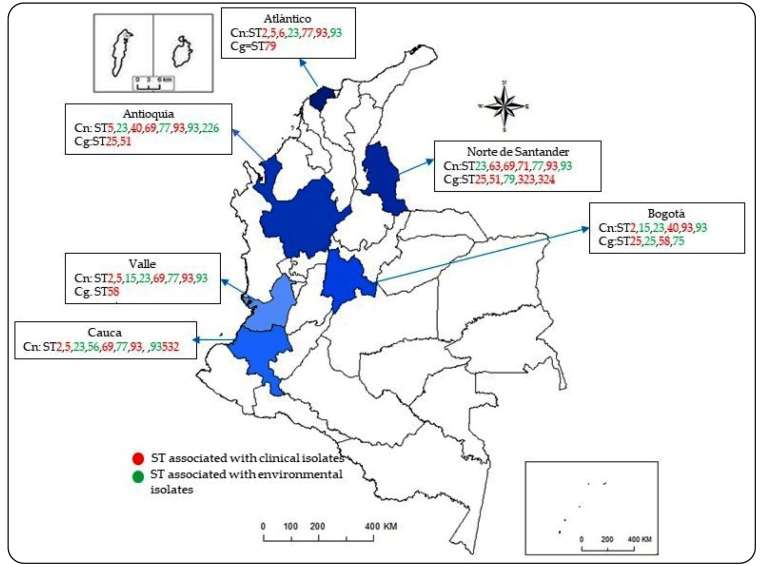
Geographical distribution of sequence types (STs) of clinical and environmental isolates of the *Cryptococcus neoformans* and *Cryptococcus gattii* species complexes recovered in Colombia. Data in red correspond to clinical isolates and those in green correspond to environmental isolates. **ST:** sequence type; **Cn:** *Cryptococcus neoformans;* **Cg:** *Cryptococcus gattii.*

Regarding the association between ST and geographical origin of the strain, *C. neoformans* ST93 was present in six departments in clinical and environmental samples, and ST77 in five departments (Antioquia, Atlántico, Cauca, Valle, and Nte. Santander); *C. gattii* ST25 was identified in three departments (Antioquia, Bogotá and N. Santander), and ST79 in two departments (Atlántico and Nte. Santander) (Figure 3).

## DISCUSSION

We evaluated the genetic diversity of the *C. neoformans* and *C. gattii* clinical and environmental isolates recovered in six departments in Colombia by MLST typing, and found 14 and 7 sequence types for each species among 88 and 22 isolates, respectively. Furthermore, we identified four of the high frequency sequence types globally reported in clinical and environmental isolates, namely ST93 and ST77, for *C. neoformans*, and ST25 and ST79, for *C. gattii*. This study is similar to the investigation by Beale A, et al. (2015) who reported 50 different sequences types in 230 isolates of *C. neoformans* var. *grubii* in Cape Town and Pietermaritzburg, KwaZulu-Natal, revealing a high degree of genetic diversity and variability in the isolates^26^.

This study is the first to report sequence type 532 of *C. neoformans* in a Colombian clinical isolate. This species showed less genetic variability possibly because the majority of isolates were molecular type VNI (n=86), and were associated with 13 STs.

Our data are comparable to that reported in a study by Ferreira-Paim et al. (2017) conducted in Southeastern Brazil, which described low genetic diversity among the isolates of *C. neoformans.* The most frequent STs reported were ST93, ST77, and ST23, in agreement with this study. This correlation may be because the topological and climatic characteristics of these two countries are similar^27^.

ST93 was recovered from a majority of clinical and environmental isolates in this study, and it is one of the most widely reported STs in different countries such as China, India, Indonesia, South Africa, Uganda Thailand, and Brazil, among others^18^ ^,^ ^28^ ^-^ ^30^. Furthermore, it has also been associated with high mortality in Uganda^29^.

Firacative et al., in 2019, used MLST analysis to show that in Cúcuta, a region with a significant number of cases in Colombia, isolates were highly clonal^30^. The molecular type of all 13 isolates was VGII, with ST25 being the most common (n=11). In our study, diverse species of *C. gattii* were found, and the isolates were not clonal as reported by Firacative et al. This may be because we included isolates from five different cities and three molecular types (VGI, VGII and VGIII). Although the most common sequence type for *C. gattii* in the present investigation was ST25, it was identified in three cities: Cucutá (n=4), Bogotá, and Antioquia (n=2 in each city).

In 2016, this same author^19^ characterized the genetic structure of the molecular type VGIII by MLST, in 122 clinical, environmental, and veterinary isolates from Australia, Colombia, Guatemala, Mexico, New Zealand, Paraguay, United States of America (USA), and Venezuela. A total of 37 Colombian isolates were included, and ST79 was the most frequent (n=13) for the country. In the present investigation, the molecular type VGIII (n=8) was included, and ST79 was the most common (n=6). This may indicate that several STs of *C. gattii* are in circulation in the country.

The less frequent STs for *C. neoformans* were ST5, ST6, and ST56, and for *C. gattii*, ST51, ST58 and ST75. Some of these STs are prevalent in Europe, Asia, North America, and Oceania. C*. neoformans* ST5 has been previously found in China, Japan, South Korea, East Asia, and Thailand in clinical cases, environmental samples, and even in veterinary cases in cats^31^ ^,^ ^32^. *C. gattii* ST51 has been found in Australia, China, India, Mexico, Papua New Guinea, and in the USA, in clinical, veterinary, and environmental samples^19^ ^,^ ^33^ ^-^ ^35^.

Globally and in accordance with the results of various studies, genetic structures vary depending on geographic location. The species of yeast causing cryptococcosis in East Asian populations are genetically less diverse compared to those from Europe, Africa, and North and South America^18^ ^,^ ^27^ ^,^ ^34^ ^-^ ^36^. One possible explanation for the diversity and distribution of sequence types observed in *C. neoformans* in the environment may be bird migration. In South America, the origin of *C. gattii* and possible global dispersion have been described, mainly in regions of Brazil where genetic diversity of this species has been found. The genetic diversity data were obtained using phylogenetic and recombination analyses based on AFLP and MLST)^36^.

## CONCLUSIONS

The effort to increase knowledge about the genetics of populations of *C. neoformans* and *C. gattii* lies with the appearance of specific genotypes associated with disease and dispersal of genetic populations. MLST revealed significant genotypic variations in *C. neoformans* and *C. gattii* in six departments of Colombia; however, the frequently reported STs indicate that in the country, diverse disease-causing strains are circulating in the environment. Expanding the cohort to other departments has been suggested, to continue detecting circulating strains.

In this study, we used the MLST technique for the molecular typing of *C. neoformans* and *C. gattii* isolates in Colombia. The importance of combining clinical and environmental isolates together with molecular data for the study of cryptococcosis was demonstrated in this study, and this approach was essential to identify genetic associations between types of strains.
