# Supplementary material for: Multilocus sequence typing (MLST) of clinical and environmental isolates of Cryptococcus neoformans and Cryptococcus gattii in six departments of Colombia reveals high genetic diversity
Source: Rev Soc Bras Med Trop. 2020 Sep 11;53:e20190422. doi: 10.1590/0037-8682-0422-2019 (PMC7491559; doi:10.1590/0037-8682-0422-2019)
Supplement: Supplementary file 1 [file 1678-9849-rsbmt-53-e20190422-suppl1.pdf]

SUPPLEMENTARY MATERIAL

Supplement 1. Sequence types of *Cryptococcus gattii* clinical isolates described by Lizarazo et al., in 2014 (20)

Isolates	Department	Molecular type	*CAP59*	*GPD1*	*IGS1*	*LAC1*	*PLB1*	*SOD1*	*URA5*	ST
H0058-I-3096	Antioquia	VGII	2	6	25	4	18	12	10	25
H0058-I-3286		VGI	16	5	3	5	5	32	12	51
H0058-I-3590			2	6	25	4	18	12	10	25
H0058-I-3031	Bogotá		16	11	13	19	15	34	14	58
H0058-I-2792	Norte Santander	VGII	2	6	25	4	18	12	10	25
H0058-I-2858			2	6	25	4	18	12	10	25
H0058-I-2877			2	6	25	4	18	12	10	25
H0058-I-3146			2	6	95	4	18	12	10	323
H0058-I-3151			16	5	3	5	5	32	12	51
H0058-I-3266			2	6	25	4	18	12	10	25
H0058-I-3407			2	21	25	4	41	12	2	324
H0058-I-3172	Valle		16	11	13	19	15	34	14	58
