# Supplementary material for: Multilocus sequence typing (MLST) of clinical and environmental isolates of Cryptococcus neoformans and Cryptococcus gattii in six departments of Colombia reveals high genetic diversity
Source: Rev Soc Bras Med Trop. 2020 Sep 11;53:e20190422. doi: 10.1590/0037-8682-0422-2019 (PMC7491559; doi:10.1590/0037-8682-0422-2019)
Supplement: Supplementary file 2 [file 1678-9849-rsbmt-53-e20190422-suppl2.pdf]

Supplement 2A. Clinical manifestations, risk factors, and outcomes of cryptococcosis patients in Colombia, 2005-2014.

	C. neoformans		*C. gattii*		Total
Demographic data	n=47	%	n=14	%	
Sex					
Male	37	78.3	10	21.7	47
Female	10	21.7	4	8.7	14
Clinical features					
Headache	32	69.6	12	26.1	44
Fever	26	56.5	6	13	32
Nausea and vomiting	26	56.5	5	10.9	31
Seizures	22	47.5	8	14.3	30
Meningeal signs	10	21.7	3	6.5	13
Visual alterations	8	17.4	3	6.5	11
Cough	7	15.2	1	2.2	8
Loss weight	8	17.4	2	4.3	10
Risk factors *					
HIV/AIDS	36	78.3	3	6.5	39
Evans Syndrome	1	2.2			1
Lupus	2	4.3			2
Arthritis			1	2.2	1
Outcome					
Alive	35	73.9	12	26.1	47
Dead	12	26.1	2	4.3	14

18 clinical cases do not report a risk factor*C. neoformans, Cryptococcus neoformans*; *C. gattii*, *Cryptococcus gattii*; HIV/AIDS, Human Immunodeficiency Virus/Acquired Immune Deficiency Syndrome;
