# Supplementary material for: Multilocus sequence typing (MLST) of clinical and environmental isolates of Cryptococcus neoformans and Cryptococcus gattii in six departments of Colombia reveals high genetic diversity
Source: Rev Soc Bras Med Trop. 2020 Sep 11;53:e20190422. doi: 10.1590/0037-8682-0422-2019 (PMC7491559; doi:10.1590/0037-8682-0422-2019)
Supplement: Supplementary file 3 [file 1678-9849-rsbmt-53-e20190422-suppl3.pdf]

Supplement 2B. Environmental isolates of *Cryptococcus neoformans* and *Cryptococcus gattii* from Colombia, described by species of trees and birds (for isolates from bird droppings)

Species	*C. neoformans*	*C. gattii*	Total (%)
Acacia mangium	2		2 (4.1)
Corymbia ficifolia	2	1	3 (6.1)
Eucalyptus	7	4	11 (22.4)
Guaiacum officinale	1		1 (2)
Licania tomentosa	2	3	5 (10.2)
Roystonea regia	1		1 (2)
Pinus sylvestris	1		1 (2)
Pithecellobium	1		1 (2)
Quercus robus	3		3 (6.1)
Terminalia catappa	8		8 (16.3)
Culumba livia	13		13 (26.5)
Total	41	8	49 (100)

C. neoformans, Cryptococcus neoformans; C. gattii, *Cryptococcus gattii*
