# Supplementary material for: Multilocus sequence typing (MLST) of clinical and environmental isolates of Cryptococcus neoformans and Cryptococcus gattii in six departments of Colombia reveals high genetic diversity
Source: Rev Soc Bras Med Trop. 2020 Sep 11;53:e20190422. doi: 10.1590/0037-8682-0422-2019 (PMC7491559; doi:10.1590/0037-8682-0422-2019)
Supplement: Supplementary file 4 [file 1678-9849-rsbmt-53-e20190422-suppl4.pdf]

Revista da Sociedade Brasileira de Medicina Tropical
Journal of the Brazilian Society of Tropical Medicine
Vol.:53:(e20190422): 2020
https://doi.org/10.1590/0037-8682-0422-2019

Major Article

Multilocus sequence typing (MLST) of clinical and environmental isolates of *Cryptococcus neoformans* and *Cryptococcus gattii* in six departments of Colombia reveals high genetic diversity

***Norida Vélez*[1] *and Patricia Escandón*[1]**

[1]. Grupo de Microbiología, Instituto Nacional de Salud, Bogotá, Colombia.

Abstract

Introduction: The average annual incidence of cryptococcosis in Colombia is 0.23 cases per 100,000 inhabitants in the general population, and 1.1 cases per 1000 in inhabitants with Acquired Immune Deficiency Syndrome (AIDS). In addition, the causal fungus has been isolated from the environment, with serotypes A-B and C in different regions. This study aims to determine the genetic association between clinical and environmental isolates of *C. neoformans/C. gattii* in Colombia. **Methods:** Multilocus sequence typing (MLST) was used to identify possible clones, providing information about the epidemiology, ecology, and etiology of this pathogen in Colombia. **Results:** A total of 110 strains, both clinical (n=61) and environmental (n=49), with 21 MLST sequence types (ST) of *C. neoformans* (n=14STs) and *C. gattii* (n=7STs) were identified. The STs which shared clinical and environmental isolate sources were grouped in different geographical categories; for *C. neoformans*, ST93 was identified in six departments, ST77 in five departments; and for *C. gattii*, ST25 was identified in three departments and ST79 in two. **Conclusions:** High genetic diversity was found in isolates of *C. neoformans/gattii* by MLST, suggesting the presence of environmental sources harboring strains which may be sources of infection for humans, especially in immunocompromised patients; these data contribute to the information available in the country on the distribution and molecular variability of *C. neoformans* and *C. gattii* isolates recovered in Colombia.

Keywords: *Cryptococcus neoformans*. *Cryptococcus gattii*. Multilocus Sequence Typing. Incidence. Colombia.

INTRODUCTION

Cryptococcosis is a fungal disease of worldwide distribution. Patients acquire the infection by exposure and inhalation of fungal propagules present in environmental sources. This infection is considered potentially fatal, and affects the lungs and the central nervous system[1-3] in both immunosuppressed individuals and in those with an apparently intact immune system[4,5]. In Colombia, the annual incidence for this infection is 0.23 cases per 100.000 inhabitants in the general population and 1.1 cases per 1000 inhabitants in Acquired Immune Deficiency Syndrome (AIDS) patients (period 1997-2013)[3].

Corresponding author: Patricia Escandón.
e-mail: pescandon@ins.gov.co
https://orcid.org/0000-0003-3029-118X
Received 9 September 2019
Accepted 29 May 2020

Although different taxonomic classifications have been proposed to categorize the etiological agent of the disease, Hagen et al. have suggested that different molecular types should be considered as independent species[6]. This suggestion has not been fully accepted by the scientific community[7]. In the present investigation, we refer to isolates as the *C. neoformans* species complex and the *C. gattii* species complex[7]. Cryptococcosis is caused by the *C. neoformans* species complex and the *C. gattii* species complex[7]. The first species consists of two varieties, *C. neoformans* var. *grubii* (serotype A) and *C. neoformans* var. *neoformans* (serotype D). In addition to the hybrid AD serotype, the species has a worldwide distribution and preferentially affects immunocompromised individuals, mainly those infected with the human immunodeficiency virus (HIV)[2-4]. In the environment, it has been associated with bird excreta, especially from soils contaminated with pigeon (*Columba livia*) droppings[8]. The second causative agent of cryptococcosis, the *C. gattii* species complex, comprises serotypes B and C, which can be found in the

environment in decaying plant material (hollows, leaf, bark, flowers, soil, fruit) from different trees (*Eucalyptus* spp., acacias, *Ficus* spp., and *Terminalia catappa*) in various regions of the world[9,10]. It is found mainly in tropical, subtropical, and temperate regions[11-13]. Currently, several interspecies hybrids have been described between serotypes BD and AB[2,14].

Many molecular techniques have been applied in the epidemiological study of *C. neoformans* and *C. gattii* isolates. The most common techniques are PCR fingerprinting[15], restriction fragment length polymorphism (RFLP) of the *PLB1* and *URA5* genes[15], amplified fragment length polymorphism (AFLP)[16], and the most recently developed technique of multilocus sequence typing (MLST)[17,18]. For genotyping *Cryptococcus* species using MLST, six conserved genes (*CAP59*, *GPD1*, *LAC1*, *PLB1*, *SOD1* and *URA5*) and the intergenic region IGS1 are used. MLST has a high discriminatory power for the genotyping of isolates to determine clonality. It is also highly discriminatory for a large number of pathogens. MLST directly measures changes in the sequence of a series of conserved genes, characterizes isolates by allelic profiles, and is an excellent tool for taxonomic characterization at the molecular level[17]. However, more robust techniques such as whole genome sequencing (WGS) allow for the detection of differences between the molecular types at the genomic level[19]. In Colombia, studies have been carried out describing the importance and potential relationship between clinical and environmental isolates using molecular typing techniques such as PCR fingerprinting and *URA5*-RFLP[10,15,20], and have contributed to the knowledge about the epidemiology of these pathogens.

Although the results from previous studies are very important and have contributed to the knowledge about the epidemiology of these pathogens, the genetic diversity of the Colombian isolates is suspected to be more diverse. Therefore, this study aims to determine the genetic relationship between clinical and environmental isolates of the *C. neoformans* species complex and the *C. gattii* species complex in Colombia using MLST. In addition, identifying possible clones and a more precise association between clinical and environmental strains will provide important information about the epidemiology, ecology, and etiology of this pathogen in Colombia.

METHODS

Study areas and biological material

A set of 88 *C. neoformans* species complex isolates (clinical: n=47; environmental: n=41) and 22 *C. gattii* species complex isolates (clinical: n=14; environmental: n=8) were included. These isolates which were recovered between 2005-2014 were available in the strain bank of the Microbiology Group at the Instituto Nacional de Salud, recovered in the departments of Antioquia (18.3%), Atlántico (14.6%), Bogotá DC (15.6%), Cauca (13.7%), Norte de Santander (20.8%), and Valle (17.4%), and were previously typed by PCR fingerprinting or RFLP of the *URA5* gene. Isolates were preserved in 10% glycerol at -70 °C. Of these isolates, 86 (78%) belonged to *C. neoformans* molecular type VNI, two (1.8%) belonged to *C. neoformans* molecular type VNII; four (3.6 %) isolates belonged to *C. gattii* molecular type VGI, 10 (9.2%) belonged to VGII, and 8 (7.3%) belonged to the VGIII molecular type. From these, 12 *C. gattii* isolates of clinical origin were previously typed by Lizarazo J, et al. in 2014[21]. Thus, a total of 110 isolates were studied (**Supplementary material 1**).

Patient data

Of a total of 61 clinical isolates (*C. neoformans*: n=47; *C. gattii*: n=14), 78.3% were isolated from men. The mean age was 42 years with a minimum of 18 years and a maximum of 82 years. The most common symptom was headache in 69.6% of the cases, followed by fever and nausea in 56.5%, while confusion, visual alterations, cough, and weight loss were also observed in a small percentage of patients; 70.4% of the patients presented with at least one risk factor, with HIV/AIDS being the most common. Of these cases, 16.6% were diagnosed concurrently with AIDS and cryptococcosis. A total of 88.3% were new cases, and 11.2% were relapses; 23.0% of the patients died (**Supplementary material 2a**).

Environmental data

Forty-nine environmental isolates were selected (*C. neoformans:* n=41; *C. gattii*: n=8); 73.5% (n=36) were recovered from 10 different types of trees, and 26.5% (n=13) were recovered from *Columba livia* droppings (**Supplementary material 2b**).

Molecular Analysis

a) Genomic DNA extraction was performed as previously described by Casali A, et al. (2003)[22]. Briefly, *C. neoformans* and *C. gattii* were plated on yeast extract-peptone-dextrose (YEPD) agar for 48 hours at 27 °C; 10 µl of yeast cells was placed in an Eppendorf tube using an inoculation loop, and incubated at -20 °C for one hour. The cells were then suspended in 500 µl of lysis buffer (10 mM Tris, pH 7.5, 1 mM EDTA, pH 8.0, and 1% SDS) and incubated at 65 °C for one hour; 500 µl of phenol:chloroform:isoamyl alcohol (25:24:1) was added, and the sample was centrifuged for 15 minutes at 13,000 rpm. The supernatant was transferred to a new tube, and an equal volume of isopropanol was added. The DNA was precipitated at -20 °C for one hour, and centrifuged for 15 minutes at 4 °C at 13,000 rpm. The DNA was then precipitated with 70% ethanol and centrifuged again for 15 minutes at 4 °C at 13,000 rpm, and subsequently dried at room temperature. The samples were resuspended in 5 µl Tris-EDTA (TE) buffer and stored at 4 °C.

b) MLST typing: Typing of the isolates was performed using the International Society for Human and Animal Mycology (ISHAM) consensus MLST scheme of seven genetic loci: *CAP59, GPD1,* IGS1*, LAC1, PBL1, SOD1,* and *URA5*[17], with minor modifications. Individual PCRs were performed in a final volume of 20 µl and a volume of 2 µl of DNA was added at a concentration of 1 ng/µl for the amplification of *CAP59*, *SOD1*, IGS1, and *GPD1,* and a volume of 5 µl of DNA at a concentration of 5 ng/µl for amplifying the *LAC1*, *PLB1,* and *URA5* genes.

PCR products were purified and sequenced commercially by the sequencing service provider Macrogen, Inc. Sequences were analyzed using the Sequencher Software 5.2 (Gene Codes Corporation, MI, USA). Six reference strains were used: WM148 (VNI-CBS10085), WM626 (VNII-CBS10086), WM179 (VGI-CBS10078), WM178 (VGII-CBS10082), WM175 (VGIII-CBS10081), and WM779 (VGIV-CBS10101)[15]. Dendrograms were created with the Mega 5.0 software, using the

individual locus sequences and the concatenated sequences[23]. The evolutionary history was derived using the maximum likelihood method based on the Jukes-Cantor model, and bootstrap values were displayed for each branch (1000 repetitions). Allele types and combined sequence types were assigned using the ISHAM consensus database[24]. The data were tabulated using Microsoft Excel®. Additionally, *C. gattii* sequences reported previously by Lizarazo J, et al. in 2014[21] were included to increase the robustness of the analysis.

Genetic diversity of isolates was determined by using the DnaSP v5 software; this variability was extracted from concatenated sequences associated with genes, department, molecular type, and origin (clinical or environmental)[25], to detect genetic polymorphism levels. The distribution was determined by calculating the haplotype (gene) diversity, nucleotide diversity (π) (the average number of nucleotide differences per site between two sequences), and θ indexes (per site, as an indicator of mutation rate per nucleotide site per generation), calculated from Eta (h) (the total number of mutations and "S", the number of segregating/polymorphic sites). Each index was reported with the corresponding standard deviation. The π indexes for each set of data were compared to identify the category with the greatest diversity.

RESULTS

A total of 98 isolates were typed by MLST (*C. neoformans*: n=88; *C. gattii*: n=10); additionally, 12 clinical isolates of *C. gattii* sequences reported previously by Lizarazo J, et al. in 2014 were included[21]. Twenty-one STs were identified, and 13 STs were assigned to the molecular type VNI and one ST to the *C. neoformans* molecular type VNII; three STs were assigned to molecular type VGII, and two STs each were assigned to the VGI and VGIII molecular types (**Table 1**). The genetic associations among the

TABLE 1: Sequence types of *Cryptococcus neoformans* and *Cryptococcus. gattii* in clinical and environmental isolates from Colombia.

		Departments						
**Molecular type**	**ST**	**Antioquia**	**Atlántico**	**Bogotá**	**Cauca**	**Nte. Santander**	**Valle**	**Total**
**Environmental**								
VNI	15	-	-	1	-	-	1	2
	23	4	4	1	1	1	2	13
	56	-	-	-	1	-	-	1
	77	1	-	-	5	1	4	11
	93	2	5	2	2	1	1	13
	226	1	-	-	-	-	-	1
VGII	25	-	-	1	-	-	-	1
VGIII	75	-	-	2	-	-	-	2
	79	-	-	-	-	5	-	5
**Clinical**								
VNI	2	-	2	2	1	-	1	6
	5	1	1	-	1	-	2	5
	6	-	1	-	-	-	-	1
	63	-	-	-	-	1	-	1
	69	1	-	-	1	1	2	5
	71	-	-	-	-	1	-	1
	77	-	1	-	-	-	-	1
	93	6	1	5	3	4	5	24
	532	-	-	-	1	-	-	1
VNII	40	1	-	1	-	-	-	2
VGI	51	1	-	-	-	1	-	2
	58		-	1	-	-	1	2
VGII	25	2	-	1	-	4	-	7
	323	-	-	-	-	1	-	1
	324	-	-	-	-	1	-	1
VGIII	79	-	1	-	-	-	-	1
	**Total**	20	16	17	16	22	19	**110**

ST: sequence type.

isolates for *C. neoformans* and *C. gattii* are shown in **Figures 1 and 2**, respectively. The MLST data of sequences of identified alleles were deposited in the GenBank database (**Supplementary material 3**).

In 88 clinical and environmental isolates of *C. neoformans,* 14 different STs were identified, the most frequent of which was ST93 (42%), followed by ST23 (14.7%), ST77 (13.6%), ST2 (6.8%), ST5, ST6, ST15, ST40, ST56, ST63, ST69, ST71, and ST226. ST532, a novel *C. neoformans* ST was identified, and this is the first report of this ST worldwide. Of the 22 isolates of *C. gattii,* seven different STs were identified; the most frequent ST was ST25 (36.3%), followed by ST79 (27.2%) and, in lesser proportions, ST51, ST58, ST75, ST323, and ST324. **Table 1** shows the STs found in both the clinical and environmental isolates.

Diversity indexes were also calculated per species, department, origin, and molecular type. It was found that the haplotype diversity in *C. neoformans* was 14 and in *C. gattii* it was 11; the nucleotide diversity index was higher in *C. gattii* (Pi= 0.868) than in *C. neoformans* (Pi= 0.779) (**Supplementary material 3)**. The diversity index calculated by departments showed that Cauca and Valle presented with greater diversity of haplotypes for *C. neoformans* (Hd=0.833 and 0.8443, respectively), and Bogotá and Norte de Santander for *C. gattii* (Hd=0.8 and 0.818 respectively). The diversity by type of origin (clinical or environmental) did not vary between the two species. The haplotypic diversity for *C. gattii* was higher in VGI and VGIII (Hd= 0.667 and 0.607 respectively), when compared to VGII (Hd= 0.378) **(Supplementary material 4)**.

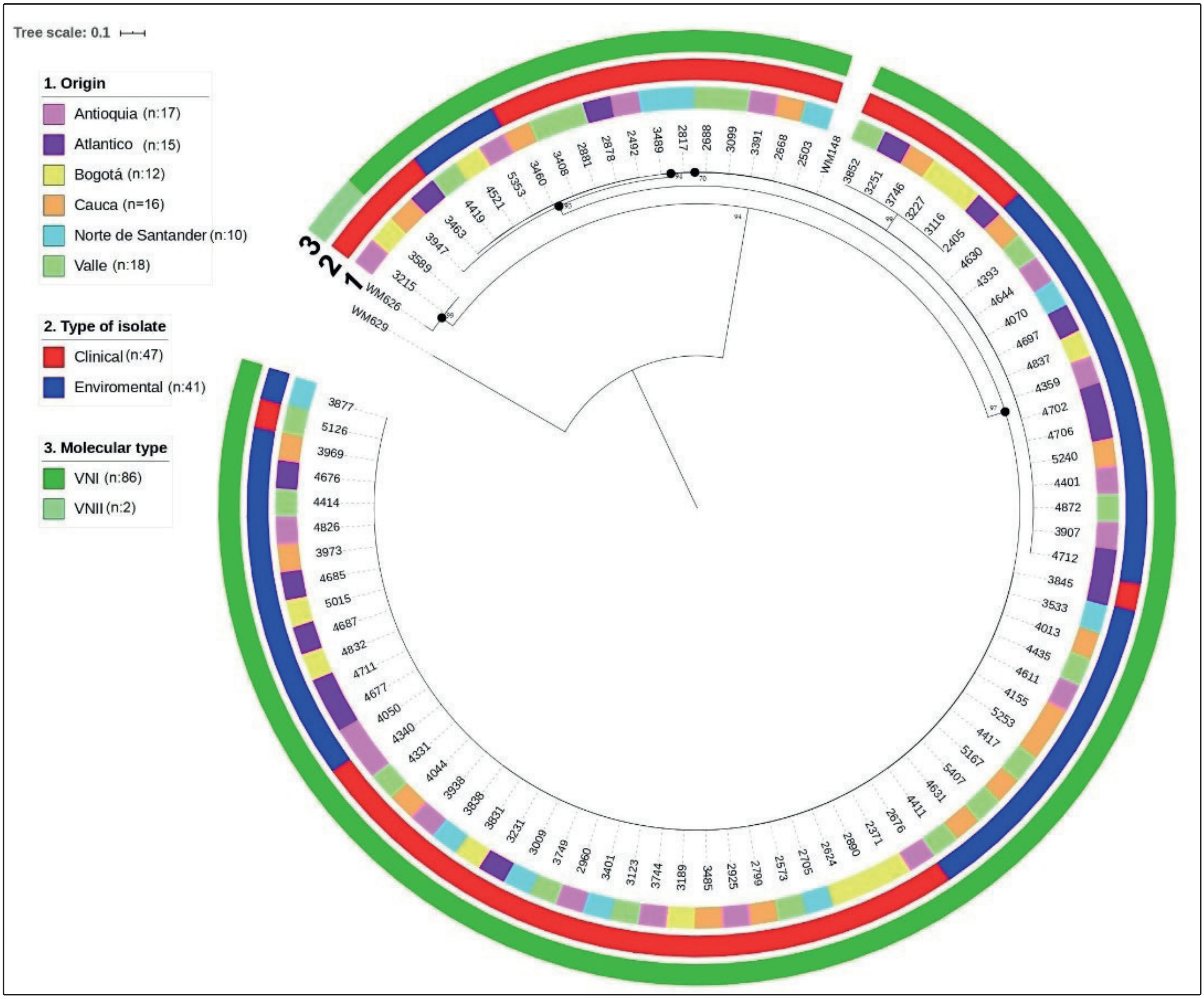

FIGURE 1: Phylogenetic analysis of 88 clinical and environmental isolates of *C. neoformans*. The evolutionary history was derived using the maximum likelihood method based on the Jukes-Cantor model using concatenated nucleotide sequences of 7 loci and a representative for each multilocus sequence typing (MLST) sequence type. Bootstrap values are shown for each branch (1000 repetitions).

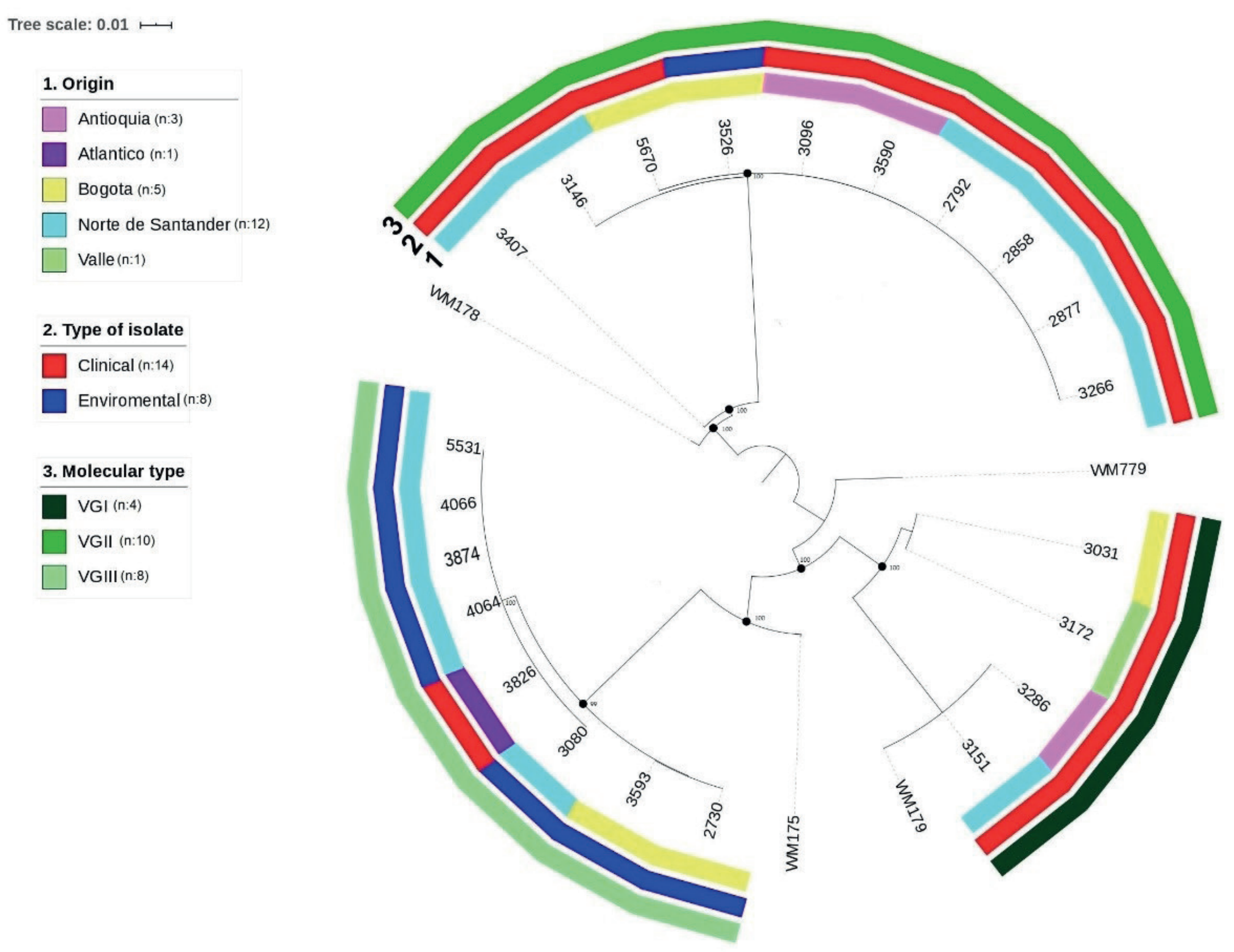

FIGURE 2: Phylogenetic analysis of 21 clinical and environmental isolates of *C. gattii*. The evolutionary history was derived using the maximum likelihood method based on the Jukes-Cantor model using concatenated nucleotide sequences of 7 loci, and a representative for each multilocus sequence typing (MLST) sequence type. Bootstrap values are shown for each branch (1000 repetitions).

Regarding the association between ST and geographical origin of the strain, *C. neoformans* ST93 was present in six departments in clinical and environmental samples, and ST77 in five departments (Antioquia, Atlántico, Cauca, Valle, and Nte. Santander); *C. gattii* ST25 was identified in three departments (Antioquia, Bogotá and N. Santander), and ST79 in two departments (Atlántico and Nte. Santander) (**Figure 3**).

DISCUSSION

We evaluated the genetic diversity of the *C. neoformans* and *C. gattii* clinical and environmental isolates recovered in six departments in Colombia by MLST typing, and found 14 and 7 sequence types for each species among 88 and 22 isolates, respectively. Furthermore, we identified four of the high frequency sequence types globally reported in clinical and environmental isolates, namely ST93 and ST77, for *C. neoformans*, and ST25 and ST79, for *C. gattii*. This study is similar to the investigation by Beale A, et al. (2015) who reported 50 different sequences types in 230 isolates of *C. neoformans* var. *grubii* in Cape Town and Pietermaritzburg, KwaZulu-Natal, revealing a high degree of genetic diversity and variability in the isolates[26].

This study is the first to report sequence type 532 of *C. neoformans* in a Colombian clinical isolate. This species showed less genetic variability possibly because the majority of isolates were molecular type VNI (n=86), and were associated with 13 STs.

Our data are comparable to that reported in a study by Ferreira-Paim et al. (2017) conducted in Southeastern Brazil, which described low genetic diversity among the isolates of *C. neoformans.* The most frequent STs reported were ST93, ST77, and ST23, in agreement with this study. This correlation may be because the topological and climatic characteristics of these two countries are similar[27].

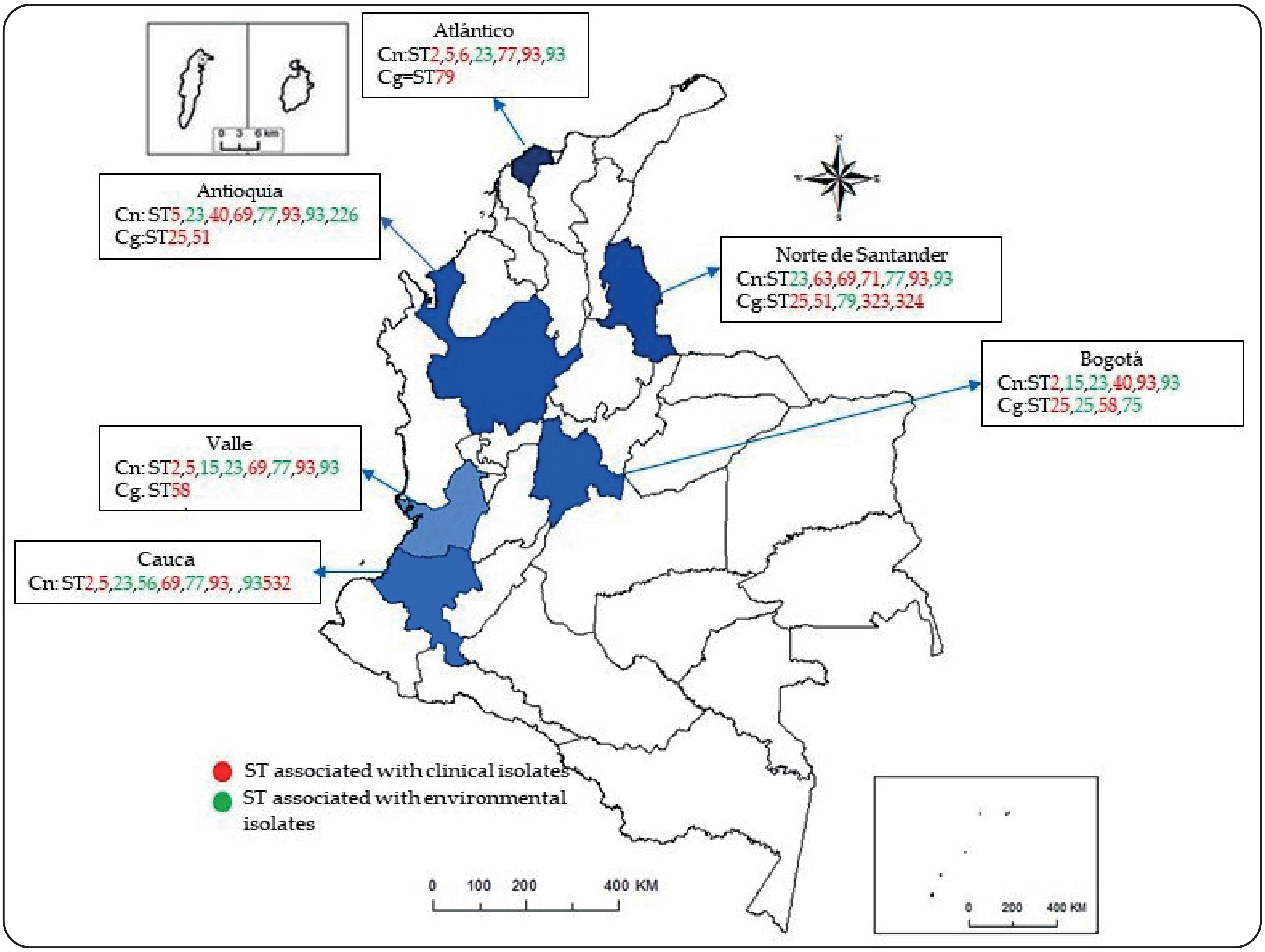

FIGURE 3: Geographical distribution of sequence types (STs) of clinical and environmental isolates of the *Cryptococcus neoformans* and *Cryptococcus gattii* species complexes recovered in Colombia. Data in red correspond to clinical isolates and those in green correspond to environmental isolates. **ST:** sequence type; **Cn:** *Cryptococcus neoformans;* **Cg:** *Cryptococcus gattii.*

ST93 was recovered from a majority of clinical and environmental isolates in this study, and it is one of the most widely reported STs in different countries such as China, India, Indonesia, South Africa, Uganda Thailand, and Brazil, among others[18,28-30]. Furthermore, it has also been associated with high mortality in Uganda[29].

Firacative et al., in 2019, used MLST analysis to show that in Cúcuta, a region with a significant number of cases in Colombia, isolates were highly clonal[30]. The molecular type of all 13 isolates was VGII, with ST25 being the most common (n=11). In our study, diverse species of *C. gattii* were found, and the isolates were not clonal as reported by Firacative et al. This may be because we included isolates from five different cities and three molecular types (VGI, VGII and VGIII). Although the most common sequence type for *C. gattii* in the present investigation was ST25, it was identified in three cities: Cucutá (n=4), Bogotá, and Antioquia (n=2 in each city).

In 2016, this same author[19] characterized the genetic structure of the molecular type VGIII by MLST, in 122 clinical, environmental, and veterinary isolates from Australia, Colombia, Guatemala, Mexico, New Zealand, Paraguay, United States of America (USA), and Venezuela. A total of 37 Colombian isolates were included, and ST79 was the most frequent (n=13) for the country. In the present investigation, the molecular type VGIII (n=8) was included, and ST79 was the most common (n=6). This may indicate that several STs of *C. gattii* are in circulation in the country.

The less frequent STs for *C. neoformans* were ST5, ST6, and ST56, and for *C. gattii*, ST51, ST58 and ST75. Some of these STs are prevalent in Europe, Asia, North America, and Oceania. *C. neoformans* ST5 has been previously found in China, Japan, South Korea, East Asia, and Thailand in clinical cases, environmental samples, and even in veterinary cases in cats[31,32]. *C. gattii* ST51 has been found in Australia, China, India, Mexico, Papua New Guinea, and in the USA, in clinical, veterinary, and environmental samples[19,33-35].

Globally and in accordance with the results of various studies, genetic structures vary depending on geographic location. The species of yeast causing cryptococcosis in East Asian populations

are genetically less diverse compared to those from Europe, Africa, and North and South America[18,27,34-36]. One possible explanation for the diversity and distribution of sequence types observed in *C. neoformans* in the environment may be bird migration. In South America, the origin of *C. gattii* and possible global dispersion have been described, mainly in regions of Brazil where genetic diversity of this species has been found. The genetic diversity data were obtained using phylogenetic and recombination analyses based on AFLP and MLST)[36].

CONCLUSIONS

The effort to increase knowledge about the genetics of populations of *C. neoformans* and *C. gattii* lies with the appearance of specific genotypes associated with disease and dispersal of genetic populations. MLST revealed significant genotypic variations in *C. neoformans* and *C. gattii* in six departments of Colombia; however, the frequently reported STs indicate that in the country, diverse disease-causing strains are circulating in the environment. Expanding the cohort to other departments has been suggested, to continue detecting circulating strains.

In this study, we used the MLST technique for the molecular typing of *C. neoformans* and *C. gattii* isolates in Colombia. The importance of combining clinical and environmental isolates together with molecular data for the study of cryptococcosis was demonstrated in this study, and this approach was essential to identify genetic associations between types of strains.

ACKNOWLEDGMENTS

We thank Dr. Carolina Firacative for critically revising the manuscript and Marina Muñoz for her help in performing the required analyses.

FINANCIAL SUPPORT

Departamento Administrativo de Ciencia, Tecnología e Innovación, Colciencias, grant 2011-3600115683.

AUTHORS' CONTRIBUTIONS

PA: Writing the proposal for the major grant, experimental work, funding, and writing and critical review of the paper; **NV:** Experimental work, data analysis, and writing of the paper.

CONFLICT OF INTERESTS

The authors declare that there are no conflicts of interests.

REFERENCES

1. Cabral P, Wood L. Animals and human beings as reservoirs for human *Cryptococcus neoformans* infection. Rev Ibe-roam Micol. 1999; 16: 77-81.
2. Bovers M, Hagen F, Kuramae E, Hoogveld H, Dromer F, Germain G, et al. AIDS patient death caused by novel *Cryptococcus neoformans* × *C. gattii* hybrid. Emerg Infect Dis. 2008;14(7):1105-08.
3. Escandon P, Lizarazo J, Agudelo C, Castañeda E. Cryptococcosis in Colombia: Compilation and analysis of Data from Laboratory-Based Surveillance. J Fungi. 2018;4(1):32.
4. Mesquita M, Martins F, RibeiroT, Magalhães M, Silva E, Chagas M. Cryptococcosis, a risk for immunocompromised and immunocompetent individuals. Open Epi. 2013;6:9-17.
5. Massimo C. Global Molecular Epidemiology of *Cryptococcus neoformans* and *Cryptococcus gattii:* An Atlas of the Molecular Types. Scientifica. 2013:1-23.
6. Hagen F, Khayhan K, Theelen B, Kolecka A, Polacheck I, Sionov E, et al. Recognition of seven species in the *Cryptococcus gattii*/*Cryptococcus neoformans* species complex. Fungal Genet Biol. 2015;78:16-48.
7. Kwon-Chung KJ, Bennett JE, Wickes BL, Meyer W, Cuomo CA, Wollenburg KR, et al. The Case for Adopting the "Species Complex" Nomenclature for the Etiologic Agents of Cryptococcosis. mSphere. 2017;2(1). pii: e00357-16. doi: 10.1128/mSphere.00357-16.
8. Soares MC, Paula CR, Dias AL, Caseiro MM, Costa SO. Environmental strains of *Cryptococcus neoformans* var. *grubii* in the city of Santos, SP, Brazil. Rev Inst Med Trop Sao Paulo. 2005;47(1):31-6.
9. Girish C, Prabu D, Mitani H. Environmental isolation of *Cryptococcus neoformans* and *Cryptococcus gattii* from living trees in Guindy National Park, Chennai, South India. Mycoses. 2010;53(3):262-4.
10. Firacative C, Torres G, Rodríguez M, Escandón P. Primer aislamiento ambiental de *Cryptococcus gattii* de serotipo B, en Cúcuta, Colombia. Biomedica. 2011;31:118-23.
11. Springer D, Chaturvedi V. Projecting global occurrence of *Cryptococcus gattii.* Emer Infect Dis. 2010;16(1):14-20.
12. Kidd E, Hagen F, Tscharke R. A rare genotype of *Cryptococcus gattii* caused the Cryptococcosis outbreak on Vancouver Island (British Columbia, Canada). PNAS. 2004;101(49):17258-63.
13. Byrnes E, Bildfell R, Frank S, Mitchell T, Marr K, Heitman J. Molecular evidence that the range of the Vancouver Island outbreak of *Cryptococcus gattii* infection has expanded into the Pacific Northwest in the United States. J. Infect Dis. 2009;199(7):1081-6.
14. Bovers M, Hagen F, Kuramae E, Diaz M, Spanjaard L, Dromer F, et al. Unique hybrids between fungal pathogens *Cryptococcus neoformans* and *Cryptococcus gattii*. FEMS Yeast Res. 2006;6(4):599- 607.
15. Meyer W, Castañeda A, Jackson S, Castañeda E, Huynnh M. Molecular typing of Ibero American *Cryptococcus neoformans* isolates. Emer Infect Dis. 2003;9(2):189-95.
16. Boekhout T, Theelen B, Diaz M. Hybrid genotypes in the pathogenic *yeast Cryptococcus neoformans.* Microbiology. 2001;147(Pt 4):891-907.
17. Meyer W, Aanensen D, Boekhout T. Consensus multi-locus sequence typing scheme for *Cryptococcus neoformans* and *Cryptococcus gattii*. Med Mycol. 2009;47(6):561-70.
18. Simwami S, Khayhan K, Henk D. Low diversity *Cryptococcus neoformans* variety *grubii* multilocus sequence types from Thailand are consistent with an ancestral African origin. PLoS Pathogens. 2011;7(4):e1001343.
19. Firacative C, Roe C, Malik R, Ferreira-Paim K, Escandón P, Sykes J, et al. MLST and Whole-Genome-Based Population Analysis of *Cryptococcus gattii* VGIII Links Clinical, Veterinary and Environmental Strains, and Reveals Divergent Serotype Specific Sub-populations and Distant Ancestors. PLoS Negl Trop Dis. 2016;10(8): e0004861.
20. Escandón P, Sánchez A, Martínez M, Meyer W, Castañeda E. Molecular epidemiology of clinical and environmental isolates of the *Cryptococcus neoformans* species complex reveals a high genetic diversity and the presence of the molecular type VGII mating type a in Colombia. FEMS Yeast Res. 2006;6(4):625-35.
21. Lizarazo J. Escandon P, Agudelo C, Firacative C, Meyer W, Castañeda E. Retrospective study of the epidemiology and clinical manifestations of *Cryptococcus gattii* infections in Colombia from 1997-2011. PLoS Negl Trop Dis. 2014;8(11):e3272.

22. Casali A, Goulart L, Silva L, Ribeiro A, Amaral A, Meyer W, et al. Molecular typing of clinical and environmental *Cryptococcus neoformans* isolates in the Brazilian state Rio Grande do Sul. FEMS Yeast Res. 2003;3(4):405-15.
23. Kumar S, Stecher G, Tamura K. MEGA7: Molecular Evolutionary Genetics Analysis Version 7.0 for Bigger Datasets. Mol Biol Evol. 2016;33(7):1870-4.
24. Fungal mlst database. Available in: http://mlst.mycologylab.org/. Consulted on 2017.02.25.
25. Librado P, Rozas J. DnaSP v5: a software for comprehensive analysis of DNA polymorphism data. Bioinformatics. 2009;25(11):1451-2.
26. Beale A, Sabiiti W, Robertson E, Fuentes K, O'Hanlon S, Jarvis J, et al. Genotypic diversity is associated with clinical outcome and phenotype in Cryptococcal meningitis across Southern Africa. PLoS Negl Trop Dis. 2015;9(6):e0003847.
27. Ferreira-Paim K, Andrade-Silva L, Fonseca F, Ferreira T, Mora D, Andrade-Silva J, et al. MLST-Based Population Genetic Analysis in a Global Context Reveals Clonality amongst *Cryptococcus neoformans* var. *grubii* VNI Isolates from HIV Patients in Southeastern Brazil. PLoS Negl Trop Dis. 2017;11(1):e0005223.
28. Dou HT1, Xu YC, Wang HZ, Li TS. Molecular epidemiology of *Cryptococcus neoformans* and *Cryptococcus gattii* in China between 2007 and 2013 using multilocus sequence typing and the DiversiLab system. Eur J Clin Microbiol Infect Dis. 2015;34(4):753-62.
29. Wiesner DL, Moskalenko O, Corcoran JM, McDonald T, Rolfes MA, Meya DB, et al. Cryptococcal genotype influences immunologic response and human clinical outcome after meningitis. MBio. 2012;3.
30. Firacative C, Torres G, Meyer W and Escandón P. Clonal Dispersal of *Cryptococcus gattii* VGII in an Endemic Region of Cryptococcosis in Colombia. J Fungi (Basel). 2019;5(2):32.
31. Danesi P, Firacative C, Cogliati M, Otranto D, Capelli G, Meyer W. Multilocus Sequence Typing (MLST) and M13 PCR fingerprinting revealed heterogeneity amongst *Cryptococcus* species obtained from Italian veterinary isolates. FEMS Yeast Res. 2014;14(6):897-909.
32. Singer L, Meyer W, Firacative C, Thompson G, Samitz E, Sykesf J. Antifungal drug susceptibility and phylogenetic diversity among *Cryptococcus* isolates from dogs and cats in North America. J Clin Microbiol. 2014;52(6):2061-70.
33. Desnos-Ollivier M, Patel S, Raoux-Barbot D, Heitman J, Dromer F. Cryptococcosis serotypes impact outcome and provide evidence of *Cryptococcus neoformans* speciation. Mbio. 2015;6(3):e00311-15.
34. Mihara T1, Izumikawa K, Kakeya H, Ngamskulrungroj P, Umeyama T, Takazono T. Multilocus sequence typing of *Cryptococcus neoformans* in non-HIV associated Cryptococcosis in Nagasaki**,** Japan. Med Mycol. 2013;51(3):252-60.
35. Umeyama T1, Ohno H, Minamoto F, Takagi T, Tanamachi C, Tanabe K. Determination of epidemiology of clinically isolated *Cryptococcus neoformans* strains in Japan by multilocus sequence typing. Jpn J Infect Dis. 2013;66(1):51-5.
36. Souto A, Bonfietti L, Ferreira-Paim K, Trilles L, Martins M, Ribeiro-Alves M, et al. Population Genetic Analysis Reveals a High Genetic Diversity in the Brazilian *Cryptococcus gattii* VGII Population and Shifts the Global Origin from the Amazon Rainforest to the Semi-arid Desert in the Northeast of Brazil. PLoS Negl Trop Dis. 2016;10(8):e0004885.

OPEN ACCESS
https://creativecommons.org/licenses/by/4.0/

SUPPLEMENTARY MATERIAL

Supplement 1. Sequence types of *Cryptococcus gattii* clinical isolates described by Lizarazo et al., in 2014 (20)

Isolates	Department	Molecular type	*CAP59*	*GPD1*	*IGS1*	*LAC1*	*PLB1*	*SOD1*	*URA5*	ST
H0058-I-3096	Antioquia	VGII	2	6	25	4	18	12	10	25
H0058-I-3286		VGI	16	5	3	5	5	32	12	51
H0058-I-3590			2	6	25	4	18	12	10	25
H0058-I-3031	Bogotá		16	11	13	19	15	34	14	58
H0058-I-2792	Norte Santander	VGII	2	6	25	4	18	12	10	25
H0058-I-2858			2	6	25	4	18	12	10	25
H0058-I-2877			2	6	25	4	18	12	10	25
H0058-I-3146			2	6	95	4	18	12	10	323
H0058-I-3151			16	5	3	5	5	32	12	51
H0058-I-3266			2	6	25	4	18	12	10	25
H0058-I-3407			2	21	25	4	41	12	2	324
H0058-I-3172	Valle		16	11	13	19	15	34	14	58

Supplement 2A. Clinical manifestations, risk factors, and outcomes of cryptococcosis patients in Colombia, 2005-2014.

	C. neoformans		*C. gattii*		Total
Demographic data	n=47	%	n=14	%	
Sex					
Male	37	78.3	10	21.7	47
Female	10	21.7	4	8.7	14
Clinical features					
Headache	32	69.6	12	26.1	44
Fever	26	56.5	6	13	32
Nausea and vomiting	26	56.5	5	10.9	31
Seizures	22	47.5	8	14.3	30
Meningeal signs	10	21.7	3	6.5	13
Visual alterations	8	17.4	3	6.5	11
Cough	7	15.2	1	2.2	8
Loss weight	8	17.4	2	4.3	10
Risk factors *					
HIV/AIDS	36	78.3	3	6.5	39
Evans Syndrome	1	2.2			1
Lupus	2	4.3			2
Arthritis			1	2.2	1
Outcome					
Alive	35	73.9	12	26.1	47
Dead	12	26.1	2	4.3	14

18 clinical cases do not report a risk factor*C. neoformans, Cryptococcus neoformans*; *C. gattii*, *Cryptococcus gattii*; HIV/AIDS, Human Immunodeficiency Virus/Acquired Immune Deficiency Syndrome;

Supplement 2B. Environmental isolates of *Cryptococcus neoformans* and *Cryptococcus gattii* from Colombia, described by species of trees and birds (for isolates from bird droppings)

Species	*C. neoformans*	*C. gattii*	Total (%)
Acacia mangium	2		2 (4.1)
Corymbia ficifolia	2	1	3 (6.1)
Eucalyptus	7	4	11 (22.4)
Guaiacum officinale	1		1 (2)
Licania tomentosa	2	3	5 (10.2)
Roystonea regia	1		1 (2)
Pinus sylvestris	1		1 (2)
Pithecellobium	1		1 (2)
Quercus robus	3		3 (6.1)
Terminalia catappa	8		8 (16.3)
Culumba livia	13		13 (26.5)
Total	41	8	49 (100)

C. neoformans, Cryptococcus neoformans; C. gattii, *Cryptococcus gattii*

Supplement 3.

		GenBank accession No.						
	ID_strain/Allele	CAP59	GPD1	IGS1	LAC1	PBL1	SOD1	URA5
C. neoformans	H0058-I-2371	MT497126	MT507884	MT507982	MT508080	MT508276	MT508178	MT508374
	H0058-I-2878	MT497127	MT507885	MT507983	MT508081	MT508277	MT508179	MT508375
	H0058-I-2676	MT497128	MT507886	MT507984	MT508082	MT508278	MT508180	MT508376
	H0058-I-2890	MT497129	MT507887	MT507985	MT508083	MT508279	MT508181	MT508377
	H0058-I-2668	MT497130	MT507888	MT507986	MT508084	MT508280	MT508182	MT508378
	H0058-I-2624	MT497131	MT507889	MT507987	MT508085	MT508281	MT508183	MT508379
	H0058-I-2817	MT497132	MT507890	MT507988	MT508086	MT508282	MT508184	MT508380
	H0058-I-2688	MT497133	MT507891	MT507989	MT508087	MT508283	MT508185	MT508381
	H0058-I-2503	MT497134	MT507892	MT507990	MT508088	MT508284	MT508186	MT508382
	H0058-I-2705	MT497135	MT507893	MT507991	MT508089	MT508285	MT508187	MT508383
	H0058-I-2881	MT497136	MT507894	MT507992	MT508090	MT508286	MT508188	MT508384
	H0058-I-2573	MT497137	MT507895	MT507993	MT508091	MT508287	MT508189	MT508385
	H0058-I-2405	MT497138	MT507896	MT507994	MT508092	MT508288	MT508190	MT508386
	H0058-I-2799	MT497139	MT507897	MT507995	MT508093	MT508289	MT508191	MT508387
	H0058-I-2925	MT497140	MT507898	MT507996	MT508094	MT508290	MT508192	MT508388
	H0058-I-3489	MT497141	MT507899	MT507997	MT508095	MT508291	MT508193	MT508389
	H0058-I-3116	MT497142	MT507900	MT507998	MT508096	MT508292	MT508194	MT508390
	H0058-I-3215	MT497143	MT507901	MT507999	MT508097	MT508293	MT508195	MT508391
	H0058-I-3746	MT497144	MT507902	MT508000	MT508098	MT508294	MT508196	MT508392
	H0058-I-3463	MT497145	MT507903	MT508001	MT508099	MT508295	MT508197	MT508393
	H0058-I-3227	MT497146	MT507904	MT508002	MT508100	MT508296	MT508198	MT508394
	H0058-I-3589	MT497147	MT507905	MT508003	MT508101	MT508297	MT508199	MT508395
	H0058-I-3460	MT497148	MT507906	MT508004	MT508102	MT508298	MT508200	MT508396
	H0058-I-3845	MT497149	MT507907	MT508005	MT508103	MT508299	MT508201	MT508397
	H0058-I-3485	MT497150	MT507908	MT508006	MT508104	MT508300	MT508202	MT508398

H0058-I-3189	MT497151	MT507909	MT508007	MT508105	MT508301	MT508203	MT508399
H0058-I-3744	MT497152	MT507910	MT508008	MT508106	MT508302	MT508204	MT508400
H0058-I-3123	MT497153	MT507911	MT508009	MT508107	MT508303	MT508205	MT508401
H0058-I-3401	MT497154	MT507912	MT508010	MT508108	MT508304	MT508206	MT508402
H0058-I-2960	MT497155	MT507913	MT508011	MT508109	MT508305	MT508207	MT508403
H0058-I-3251	MT497156	MT507914	MT508012	MT508110	MT508306	MT508208	MT508404
H0058-I-3749	MT497157	MT507915	MT508013	MT508111	MT508307	MT508209	MT508405
H0058-I-2492	MT497158	MT507916	MT508014	MT508112	MT508308	MT508210	MT508406
H0058-I-3009	MT497159	MT507917	MT508015	MT508113	MT508309	MT508211	MT508407
H0058-I-3099	MT497160	MT507918	MT508016	MT508114	MT508310	MT508212	MT508408
H0058-I-3231	MT497161	MT507919	MT508017	MT508115	MT508311	MT508213	MT508409
H0058-I-3391	MT497162	MT507920	MT508018	MT508116	MT508312	MT508214	MT508410
H0058-I-3408	MT497163	MT507921	MT508019	MT508117	MT508313	MT508215	MT508411
H0058-I-3831	MT497164	MT507922	MT508020	MT508118	MT508314	MT508216	MT508412
H0058-I-3838	MT497165	MT507923	MT508021	MT508119	MT508315	MT508217	MT508413
H0058-I-3852	MT497166	MT507924	MT508022	MT508120	MT508316	MT508218	MT508414
H0058-I-3938	MT497167	MT507925	MT508023	MT508121	MT508317	MT508219	MT508415
H0058-I-3947	MT497168	MT507926	MT508024	MT508122	MT508318	MT508220	MT508416
H0058-I-4044	MT497169	MT507927	MT508025	MT508123	MT508319	MT508221	MT508417
H0058-I-4331	MT497170	MT507928	MT508026	MT508124	MT508320	MT508222	MT508418
H0058-I-4340	MT497171	MT507929	MT508027	MT508125	MT508321	MT508223	MT508419
H0058-I-3533	MT497172	MT507930	MT508028	MT508126	MT508322	MT508224	MT508420
H0058-I-3907	MT497173	MT507931	MT508029	MT508127	MT508323	MT508225	MT508421
H0058-I-4013	MT497174	MT507932	MT508030	MT508128	MT508324	MT508226	MT508422
H0058-I-4050	MT497175	MT507933	MT508031	MT508129	MT508325	MT508227	MT508423
H0058-I-4155	MT497176	MT507934	MT508032	MT508130	MT508326	MT508228	MT508424
H0058-I-4393	MT497177	MT507935	MT508033	MT508131	MT508327	MT508229	MT508425
H0058-I-4401	MT497178	MT507936	MT508034	MT508132	MT508328	MT508230	MT508426
H0058-I-4419	MT497179	MT507937	MT508035	MT508133	MT508329	MT508231	MT508427

H0058-I-4630	MT497180	MT507938	MT508036	MT508134	MT508330	MT508232	MT508428
H0058-I-4677	MT497181	MT507939	MT508037	MT508135	MT508331	MT508233	MT508429
H0058-I-4702	MT497182	MT507940	MT508038	MT508136	MT508332	MT508234	MT508430
H0058-I-4711	MT497183	MT507941	MT508039	MT508137	MT508333	MT508235	MT508431
H0058-I-4832	MT497184	MT507942	MT508040	MT508138	MT508334	MT508236	MT508432
H0058-I-4837	MT497185	MT507943	MT508041	MT508139	MT508335	MT508237	MT508433
H0058-I-5015	MT497186	MT507944	MT508042	MT508140	MT508336	MT508238	MT508434
H0058-I-5240	MT497187	MT507945	MT508043	MT508141	MT508337	MT508239	MT508435
H0058-I-5253	MT497188	MT507946	MT508044	MT508142	MT508338	MT508240	MT508436
H0058-I-5353	MT497189	MT507947	MT508045	MT508143	MT508339	MT508241	MT508437
H0058-I-5167	MT497190	MT507948	MT508046	MT508144	MT508340	MT508242	MT508438
H0058-I-5407	MT497191	MT507949	MT508047	MT508145	MT508341	MT508243	MT508439
H0058-I-4687	MT497192	MT507950	MT508048	MT508146	MT508342	MT508244	MT508440
H0058-I-4872	MT497193	MT507951	MT508049	MT508147	MT508343	MT508245	MT508441
H0058-I-4685	MT497194	MT507952	MT508050	MT508148	MT508344	MT508246	MT508442
H0058-I-4521	MT497195	MT507953	MT508051	MT508149	MT508345	MT508247	MT508443
H0058-I-3973	MT497196	MT507954	MT508052	MT508150	MT508346	MT508248	MT508444
H0058-I-4706	MT497197	MT507955	MT508053	MT508151	MT508347	MT508249	MT508445
H0058-I-4359	MT497198	MT507956	MT508054	MT508152	MT508348	MT508250	MT508446
H0058-I-4070	MT497199	MT507957	MT508055	MT508153	MT508349	MT508251	MT508447
H0058-I-4826	MT497200	MT507958	MT508056	MT508154	MT508350	MT508252	MT508448
H0058-I-4611	MT497201	MT507959	MT508057	MT508155	MT508351	MT508253	MT508449
H0058-I-4712	MT497202	MT507960	MT508058	MT508156	MT508352	MT508254	MT508450
H0058-I-4411	MT497203	MT507961	MT508059	MT508157	MT508353	MT508255	MT508451
H0058-I-4414	MT497204	MT507962	MT508060	MT508158	MT508354	MT508256	MT508452
H0058-I-4435	MT497205	MT507963	MT508061	MT508159	MT508355	MT508257	MT508453
H0058-I-4676	MT497206	MT507964	MT508062	MT508160	MT508356	MT508258	MT508454
H0058-I-4697	MT497207	MT507965	MT508063	MT508161	MT508357	MT508259	MT508455
H0058-I-4631	MT497208	MT507966	MT508064	MT508162	MT508358	MT508260	MT508456

	H0058-I-4644	MT497209	MT507967	MT508065	MT508163	MT508359	MT508261	MT508457
	H0058-I-4417	MT497210	MT507968	MT508066	MT508164	MT508360	MT508262	MT508458
	H0058-I-3969	MT497211	MT507969	MT508067	MT508165	MT508361	MT508263	MT508459
	H0058-I-5126	MT497212	MT507970	MT508068	MT508166	MT508362	MT508264	MT508460
	H0058-I-3877	MT497213	MT507971	MT508069	MT508167	MT508363	MT508265	MT508461
C. gattii	H0058-I-3874	MT497214	MT507972	MT508070	MT508168	MT508364	MT508266	MT508462
	H0058-I-3080	MT497215	MT507973	MT508071	MT508169	MT508365	MT508267	MT508463
	H0058-I-3826	MT497216	MT507974	MT508072	MT508170	MT508366	MT508268	MT508464
	H0058-I-5670	MT497217	MT507975	MT508073	MT508171	MT508367	MT508269	MT508465
	H0058-I-2730	MT497218	MT507976	MT508074	MT508172	MT508368	MT508270	MT508466
	H0058-I-3526	MT497219	MT507977	MT508075	MT508173	MT508369	MT508271	MT508467
	H0058-I-3593	MT497220	MT507978	MT508076	MT508174	MT508370	MT508272	MT508468
	H0058-I-4064	MT497221	MT507979	MT508077	MT508175	MT508371	MT508273	MT508469
	H0058-I-4066	MT497222	MT507980	MT508078	MT508176	MT508372	MT508274	MT508470
	H0058-I-5531	MT497223	MT507981	MT508079	MT508177	MT508373	MT508275	MT508471

C. neoformans, *Cryptococcus neoformans*; *C. gattii*, *Cryptococcus gattii*

Supplement 4. Diversity index of *C. neoformans* and *C. gattii* in clinical and environmental isolates from Colombia.

Genetic diversity	Number of sequences used	Number of polymorphic (segregating) sites (S)	Total number of mutations (Eta)	Number of haplotypes (h)	Haplotype diversity (Hd)	Nucleotide diversity (Pi)	Theta (per site) from Eta	Theta (per site) from S (ThetaW)
C. neoformans	88	86	86	14	0.779	0.00277	0.00428	0.00428
**Departament**								
Antioquia	17	67	67	7	0.75	0.00355	0.00497	0.00497
Atlántico	15	23	23	6	0.79	0.00233	0.00177	0.00177
Bogotá	12	63	63	5	0.667	0.00374	0.00524	0.00524
Cauca	16	51	51	8	0.833	0.00303	0.00385	0.00385
Norte de Santander	10	25	25	6	0.778	0.00246	0.00221	0.00221
Valle	18	28	28	7	0.843	0.00248	0.00204	0.00201
**Origin**								
Clinical	47	84	84	10	0.711	0.0033	0.00478	0.00478
Environmental	41	21	21	6	0.741	0.00216	0.00123	0.00123
**Molecular type**								
VNI	86	53	53	13	0.769	0.0023	0.00265	0.00265
VNII	2	0	0	1	0	0	0	0
C. gattii	21	1245	1313	11	0.868	0.11523	0.8619	0.08619
**Departament**								
Antioquia	3	827	827	2	0.314	0.13573	0.13573	0.13573
Atlántico	1	0	0	0	0	0	0	0
Bogotá	5	861	876	3	0.8	0.12537	0.10418	0.1024
Norte de Santander	12	1125	1160	6	0.818	0.11691	0.09586	0.09297
Valle	1	0	0	0	0	0	0	0
**Origin**								
Clinical	14	1148	1183	6	0.747	0.09843	0.09279	0.09005

Environmental	8	811	824	4	0.75	0.06394	0.07843	0.07719
**Molecular type**								
VGI	4	328	328	2	0.667	0.05324	0.04356	0.04356
VGII	10	347	347	3	0.378	0.01697	0.03	0.03
VGIII	8	306	310	3	0.607	0.01971	0.02913	0.02876
