# Supplementary material for: Multilocus sequence typing (MLST) of clinical and environmental isolates of Cryptococcus neoformans and Cryptococcus gattii in six departments of Colombia reveals high genetic diversity
Source: Rev Soc Bras Med Trop. 2020 Sep 11;53:e20190422. doi: 10.1590/0037-8682-0422-2019 (PMC7491559; doi:10.1590/0037-8682-0422-2019)
Supplement: Supplementary file 5 [file 1678-9849-rsbmt-53-e20190422-suppl5.pdf]

Supplement 4. Diversity index of *C. neoformans* and *C. gattii* in clinical and environmental isolates from Colombia.

Genetic diversity	Number of sequences used	Number of polymorphic (segregating) sites (S)	Total number of mutations (Eta)	Number of haplotypes (h)	Haplotype diversity (Hd)	Nucleotide diversity (Pi)	Theta (per site) from Eta	Theta (per site) from S (ThetaW)
C. neoformans	88	86	86	14	0.779	0.00277	0.00428	0.00428
**Departament**								
Antioquia	17	67	67	7	0.75	0.00355	0.00497	0.00497
Atlántico	15	23	23	6	0.79	0.00233	0.00177	0.00177
Bogotá	12	63	63	5	0.667	0.00374	0.00524	0.00524
Cauca	16	51	51	8	0.833	0.00303	0.00385	0.00385
Norte de Santander	10	25	25	6	0.778	0.00246	0.00221	0.00221
Valle	18	28	28	7	0.843	0.00248	0.00204	0.00201
**Origin**								
Clinical	47	84	84	10	0.711	0.0033	0.00478	0.00478
Environmental	41	21	21	6	0.741	0.00216	0.00123	0.00123
**Molecular type**								
VNI	86	53	53	13	0.769	0.0023	0.00265	0.00265
VNII	2	0	0	1	0	0	0	0
C. gattii	21	1245	1313	11	0.868	0.11523	0.8619	0.08619
**Departament**								
Antioquia	3	827	827	2	0.314	0.13573	0.13573	0.13573
Atlántico	1	0	0	0	0	0	0	0
Bogotá	5	861	876	3	0.8	0.12537	0.10418	0.1024
Norte de Santander	12	1125	1160	6	0.818	0.11691	0.09586	0.09297
Valle	1	0	0	0	0	0	0	0
**Origin**								
Clinical	14	1148	1183	6	0.747	0.09843	0.09279	0.09005

Environmental	8	811	824	4	0.75	0.06394	0.07843	0.07719
**Molecular type**								
VGI	4	328	328	2	0.667	0.05324	0.04356	0.04356
VGII	10	347	347	3	0.378	0.01697	0.03	0.03
VGIII	8	306	310	3	0.607	0.01971	0.02913	0.02876
